# A Cross-Modal Temporal Alignment Framework for Artificial Intelligence-Driven Sensing in Multilingual Risk Monitoring

**DOI:** 10.3390/s26082319

**Published:** 2026-04-09

**Authors:** Hanzhi Sun, Jiarui Zhang, Wei Hong, Yihan Fang, Mengqi Ma, Kehan Shi, Manzhou Li

**Affiliations:** 1 China Agricultural University, Beijing 100083, China; 2 National School of Development, Peking University, Beijing 100871, China

**Keywords:** cross-modal data fusion, artificial intelligence-driven sensing, multilingual semantic modeling, temporal alignment attention, cross-market generalization

## Abstract

Against the background of highly interconnected global capital markets and rapidly propagating cross-lingual information streams, traditional anomaly detection paradigms based solely on single-modality numerical time-series sensors are insufficient for forward-looking risk sensing. From the perspective of artificial intelligence-driven sensing, this study proposes a multilingual semantic–numerical collaborative Transformer framework to construct a unified multimodal financial sensing architecture for intelligent anomaly sensing and risk perception. Within the proposed sensing paradigm, multilingual texts are conceptualized as semantic sensors that continuously emit event-driven sensing signals, while market prices, trading volumes, and order book dynamics are modeled as heterogeneous numerical sensor streams reflecting behavioral market sensing responses. These heterogeneous sensors are jointly integrated through a cross-modal sensor fusion architecture. A cross-modal temporal alignment attention mechanism is designed to explicitly model dynamic lag structures between semantic sensing signals and numerical sensor responses, enabling temporally adaptive sensor-level alignment and fusion. To enhance sensing robustness, a multilingual semantic noise-robust encoding module is introduced to suppress unreliable textual sensor noise and stabilize cross-lingual semantic sensing representations. Furthermore, a semantic–numerical collaborative risk fusion module is constructed within a shared latent sensing space to achieve adaptive sensor contribution weighting and cross-sensor feature coupling, thereby improving anomaly sensing accuracy and robustness under complex multimodal sensing environments. Extensive experiments conducted on real-world multi-market financial sensing datasets demonstrate that the proposed artificial intelligence-driven sensing framework significantly outperforms representative statistical and deep learning baselines. The framework achieves a Precision of 0.852, Recall of 0.781, F1-score of 0.815, and an AUC of 0.892, while substantially improving early warning time in practical risk sensing scenarios. In cross-market transfer settings, the proposed sensing architecture maintains stable anomaly sensing performance under bidirectional domain shifts, with AUC consistently exceeding 0.86, indicating strong structural generalization across heterogeneous sensing environments. Ablation analysis further verifies that temporal sensor alignment, semantic sensor denoising, and collaborative cross-sensor risk coupling contribute independently and synergistically to the overall sensing performance. Overall, this study establishes a scalable multimodal intelligent sensing framework for dynamic financial anomaly sensing, providing an effective artificial intelligence-driven sensing solution for cross-market risk surveillance and adaptive financial signal sensing.

## 1. Introduction

In today’s interconnected global capital markets, information flows rapidly. Consequently, the driving mechanisms behind price fluctuations have undergone a profound transformation. In traditional markets, supply–demand relationships and trading behaviors primarily determined prices. However, in the contemporary digital environment, cross-lingual information streams have emerged as critical exogenous shock sources that influence market volatility [1]. International news, central bank policy statements, corporate disclosures, and social media sentiment propagate synchronously across different language spaces. These signals are rapidly amplified by algorithmic trading systems [2]. This process gradually shapes markets into a semantically event-driven dynamic regime [3]. High-frequency trading and quantitative strategies now dominate many market environments. In these contexts, abnormal fluctuations are rarely the result of endogenous evolutions in historical price structures. Instead, they are often triggered by sudden signals from semantic sensors and diffuse within a very short time horizon [4]. Therefore, constructing intelligent systems that can simultaneously perceive semantic shocks and market behaviors has become a critical issue for financial risk early warning and anomaly detection [5]. This problem is directly associated with trading security and asset management efficiency. It also exerts a significant influence on systemic risk prevention and cross-market stability. Thus, the task holds substantial practical relevance and theoretical value for intelligent financial regulation and the enhancement of global market resilience.

Traditional financial anomaly detection methods are predominantly established upon numerical time-series modeling frameworks. Early studies typically employed statistical models to characterize prices and volatility. These included linear models based on autoregressive structures and conditional heteroscedasticity models [6]. Such approaches can capture autocorrelation structures and volatility clustering phenomena to a certain extent. However, their modeling assumptions often rely on stationarity or linear structures. This renders them inadequate for complex nonlinear shock scenarios [7,8]. Under extreme market conditions or black swan events, statistical models frequently exhibit substantial performance degradation as historical patterns break down [9]. With the advancement of machine learning, feature-engineering-based anomaly detection approaches were introduced. These methods combined technical indicators, volatility features, and statistical metrics for classification or regression prediction [5]. Nevertheless, these methods remain confined to the ex post modeling of trading outcomes. They lack ex ante perception of anomaly triggers and offer limited interpretability regarding risk origins [10]. Furthermore, in the context of multi-market interdependence, single-modality numerical modeling paradigms are insufficient. They cannot capture the complex coupling relationship between semantic sensors and market fluctuations, thereby constraining detection sensitivity and early warning capability [11].

Recently, deep learning methods have introduced new breakthroughs in financial time-series modeling. Recurrent neural networks, long short-term memory networks, and transformer models based on self-attention mechanisms have demonstrated strong capabilities in modeling long-range dependencies and nonlinear structures [12]. Meanwhile, the development of multilingual pre-trained language models has enabled cross-lingual representation of signals from semantic sensors [13]. Research efforts have explored the incorporation of sentiment indices, news embeddings, and event labels to assist market prediction. However, most existing deep learning approaches rely on feature concatenation or simple attention-based fusion strategies. They directly integrate semantic sensor features and numerical features within a unified space without a systematic design for cross-modal temporal alignment mechanisms [8]. Semantic sensor signals and market fluctuations often exhibit dynamic time lags and heterogeneous propagation paths. Without effective modeling of such transmission processes, fusion models may introduce noise or produce misjudgments [11]. Additionally, significant variations in financial terminology across languages and elevated noise levels in multilingual environments may weaken model stability. This is particularly true in the absence of robust encoding mechanisms for semantic sensors. Therefore, constructing a cross-modal anomaly detection framework that simultaneously accounts for semantic shock modeling, temporal alignment, and noise suppression remains a fundamental challenge.

To address these issues, we propose a multilingual semantic–numerical collaborative sensing transformer framework. This architecture enables deep integration between semantic sensors and market behavior sensors.

The main contributions are summarized as follows.

1. A multilingual semantic–numerical collaborative sensing paradigm is proposed, in which multilingual large language models are embedded into the financial anomaly detection pipeline, facilitating the transition from purely numerical behavior detection to semantically driven risk perception and providing a novel modeling perspective for intelligent financial monitoring.
2. A cross-modal temporal alignment attention mechanism is designed, wherein learnable temporal offset parameters are introduced to characterize the dynamic lag of semantic event transmission toward market fluctuations, alleviating the misalignment between textual and price sequences and enhancing early warning capability.
3. A multilingual semantic noise-robust encoding module is constructed by incorporating semantic confidence weighting and contrastive learning mechanisms, thereby improving model stability and generalization under complex linguistic contexts and low-quality textual environments.
4. A semantic–numerical collaborative risk fusion module is developed to model the coupling relationship between semantic shock intensity and market volatility amplitude within a unified latent space; a gated fusion strategy is employed to achieve adaptive allocation of risk contributions, improving anomaly recognition accuracy and robustness.
5. Extensive experiments conducted on multi-market real-world datasets demonstrate that the proposed framework significantly outperforms traditional statistical models and deep temporal models in terms of accuracy, f1 score, AUC, and early warning time, indicating strong practical applicability.

## 2. Related Work

### 2.1. Financial Time-Series Anomaly Detection

The fundamental principle of financial time-series anomaly detection lies in statistically modeling numerical signals such as price, trading volume, and volatility to characterize the dynamic evolution structure of the market under a “normal state,” and subsequently identifying deviations from this baseline as anomalous behaviors [14]. Early studies were primarily grounded in time-series analysis theory, where linear dynamic system models were constructed through autoregressive and moving average mechanisms to forecast future trends based on historical autocorrelation structures. In parallel, conditional heteroscedasticity modeling approaches were employed to characterize volatility clustering and risk spillover effects. The underlying assumption of these methods is that market fluctuations can be regarded as the evolutionary outcome of endogenous stochastic processes, and observations that significantly deviate from model prediction intervals are classified as anomalies. Although these approaches benefit from solid theoretical foundations and relatively strong interpretability, their modeling assumptions typically rely on stationarity and linear structures, rendering them insufficient for handling structural breaks and nonlinear shocks that frequently occur in real-world financial markets. With the continuous enhancement of computational capacity and the rapid expansion of data scale, deep learning methods have gradually been introduced into the domain of financial anomaly detection [15]. Recurrent neural networks capture long- and short-term dependencies through gating mechanisms, while self-attention architectures enable the modeling of global dependencies across extended temporal horizons, thereby strengthening model fitting capability in complex dynamic environments [16]. Recent research has further extended these deep architectures to specialized financial domains; for instance, Song et al. utilized a VAE–Transformer approach to model anomalies in decentralized finance (DeFi) environments [17], while Feng et al. explored federated learning with Siamese networks to improve transaction risk discrimination [18]. In anomaly detection tasks, deep models commonly identify abnormal states through reconstruction error estimation, probabilistic distribution modeling, or classification-based discrimination. Fundamentally, these approaches remain grounded in historical numerical behavior modeling, whereby deviations from learned normal price patterns are treated as anomalies [19]. Although superior accuracy has been achieved compared with traditional statistical models, the underlying paradigm remains numerical behavior sensing. Consequently, anomalies are typically detected only after they have been reflected in price or trading volume signals, rather than being anticipated before semantic shocks are fully transmitted to the market. Under extreme market conditions or black swan events, when historical data distributions shift drastically relative to the prevailing environment, models that rely exclusively on historical numerical patterns often exhibit degraded generalization capability or even complete failure. Therefore, in the context of rapid global information dissemination, anomaly detection methods based solely on numerical sensing signals increasingly reveal limitations in forward-looking capacity and interpretability.

### 2.2. Multilingual Financial Text Analysis and Large Language Models

The fundamental objective of financial text analysis is to extract sentiment orientations, event categories, and risk signals embedded within textual content through natural language processing techniques, and to transform them into quantifiable features for market prediction and risk assessment [6]. Early research primarily concentrated on monolingual sentiment classification and keyword matching. Sentiment lexicons or supervised text classifiers were constructed to categorize news articles or corporate announcements into positive, negative, or neutral sentiments, followed by correlation analysis with market returns [20]. Although partial sentiment shock effects could be captured, expressive capacity was constrained by handcrafted features and single-language contexts, limiting applicability in cross-lingual complex semantic environments [21]. In recent years, the development of multilingual pre-trained language models has provided a technical foundation for unified cross-lingual semantic representation. Through large-scale corpus pre-training, textual data in different languages can be encoded into a shared vector space, enabling cross-lingual semantic alignment and facilitating the identification of complex event structures such as policy changes, corporate risks, and macroeconomic shocks [22]. In the financial domain, multilingual models have been utilized for cross-national news event extraction, risk signal identification, and market trend forecasting. The core concept underlying these approaches is to conceptualize textual information as a “semantic sensor,” whereby latent risks are inferred through semantic variations [23]. However, most existing studies remain confined to modeling within the textual modality, emphasizing improvements in sentiment classification accuracy or event recognition performance, while lacking deep collaborative mechanisms with numerical market signals [24]. In practical financial systems, semantic shocks are typically transmitted to price levels through complex pathways, and substantial discrepancies exist across languages in temporal scales and expressive granularity. Without unified cross-modal alignment and noise filtering mechanisms, isolated text analysis is insufficient to establish a closed-loop risk sensing framework [25]. Consequently, the systematic integration of multilingual semantic representations with market behavioral data has become a critical issue for enhancing the forward-looking capability of anomaly detection.

### 2.3. Cross-Modal Financial Intelligent Sensing Methods

Cross-modal financial intelligent sensing methods are theoretically grounded in multi-source information fusion and heterogeneous data collaborative modeling. The objective is to integrate textual semantic signals and numerical market signals within a unified representation space, thereby enhancing comprehensive perception of risk states [26]. Several studies have attempted to combine news sentiment indices, announcement embeddings, or social media indicators with price sequences through feature concatenation or simple attention mechanisms. Advanced hybrid architectures have emerged to handle these heterogeneous streams; for example, Wu and Xiang proposed an ensemble model combining VAEs, Transformers, and Graph Neural Networks (GNNs) for enterprise-level anomaly detection [27]. Typically, textual and numerical data are encoded independently, followed by concatenation or weighted summation in high-dimensional feature space, and subsequently input into downstream prediction models for anomaly identification. Although implementation complexity remains relatively low and predictive performance can be improved to a certain extent, such strategies remain fundamentally shallow fusion approaches. Beyond predictive accuracy, the robustness of such sensing systems is increasingly tied to their interpretability. As highlighted in the broader literature on data-driven predictive modeling, integrating explainable artificial intelligence (XAI) techniques is crucial for improving both the robustness and the reliability of complex predictive systems [28]. This alignment with XAI objectives is particularly relevant for financial monitoring, where understanding the causal link between a semantic shock and a subsequent market fluctuation is essential for regulatory transparency. First, along the temporal dimension, dynamic lags and multi-stage transmission pathways frequently exist between textual events and market fluctuations, whereas simple concatenation implicitly assumes perfect temporal correspondence between modalities at identical time points, thereby neglecting misalignment during event propagation [29]. Second, at the semantic level, multilingual textual data often exhibit elevated noise levels, and substantial differences may exist in how identical financial events are expressed across languages. Without robust encoding mechanisms, fusion outcomes may be adversely influenced by low-quality semantic signals [30]. Third, at the scale level, textual embeddings and numerical features differ significantly in statistical distributions and representational dimensionality. In the absence of systematic modeling, modality imbalance may arise, potentially causing the model to favor one modality and weaken overall performance. Therefore, existing cross-modal methods in anomaly detection tasks remain limited in fully exploiting the forward-looking advantages provided by semantic information.

## 3. Materials and Method

### 3.1. Data Collection

Data acquisition was conducted under a multi-source artificial intelligence-driven financial sensing framework, where heterogeneous sensing streams were constructed from both market behavior sensors and multilingual semantic sensors, as summarized in Table 1. The sensing system integrates structured numerical sensor streams that reflect real-time market microstructure dynamics and unstructured semantic sensor streams that capture cross-lingual event-driven information propagation.

The numerical sensing component was collected from historical market sensing databases and publicly accessible high-frequency matching-system sensor interfaces of major global stock exchanges, including the New York Stock Exchange (NYSE, New York, NY, USA), the NASDAQ Stock Market (NASDAQ, New York, NY, USA), the Shanghai Stock Exchange (SSE, Shanghai, China), and the Shenzhen Stock Exchange (SZSE, Shenzhen, China). The sensing horizon spans from January 2018 to December 2024, covering normal trading regimes as well as multiple extreme volatility sensing intervals. High-frequency market behavior sensors were sampled at a base temporal resolution of one minute, while tick-level transaction sensor streams and top-five-level order book depth sensor measurements were simultaneously retained to preserve microstructure-level sensing granularity. The collected sensor variables include open, high, low, and close price signals; trading volume and transaction value streams; bid–ask spread measurements; order book quoted volume signals; turnover rate; and derived volatility-sensitive sensor indicators such as moving average convergence divergence (MACD), relative strength index (RSI), and Bollinger band width. These specific signals were prioritized because market price, volume, and order-book dynamics constitute the fundamental behavioral sensing primitives from which liquidity and volatility are derived; by focusing on these core variables, the framework captures the primary market-state dynamics and execution processes that reflect real-time investor responses to exogenous shocks. Raw numerical sensor streams were batch-acquired via exchange APIs and temporally synchronized through timestamp alignment. Sensor-level preprocessing was subsequently performed, including missing-sensor imputation, abnormal sensor spike removal, and time-zone normalization, ensuring cross-market sensing consistency and structural comparability. Ultimately, a large-scale high-frequency market sensing dataset covering 1200 representative stocks and index instruments was constructed, comprising more than $4.5\times 10^{8}$ structured time-series sensor records.

The semantic sensing component was constructed from authoritative international financial information platforms, listed-company disclosure sensing channels, and publicly accessible social media sensing interfaces. English semantic sensor streams were collected from historical archives of Refinitiv (London, UK) and Bloomberg (New York, NY, USA), while Chinese semantic sensing signals were acquired from CNINFO (Shenzhen, China) and the Wind database (Wind Information Co., Ltd., Shanghai, China). In addition, public sentiment sensing streams from Twitter (San Francisco, CA, USA) and major Chinese financial forums were incorporated to capture distributed crowd-level semantic sensing dynamics. The semantic sensing window was strictly aligned with the numerical sensing horizon to ensure cross-modal temporal synchronization across heterogeneous sensors. Semantic signal acquisition followed a dual-stage sensing protocol combining keyword-triggered event sensing and financial entity-level semantic sensing. The motivation behind this hybrid design is to bridge the gap between broad macroeconomic awareness and precise firm-specific risk perception: the keyword-triggered stage provides a wide-area sensing of systemic events, while the entity-level stage enables the precise association of these events with specific financial instruments, offering a more targeted and comprehensive signal capture than single-stage approaches. Keywords were designed to capture macroeconomic policy sensors, corporate risk sensors, industry shock sensors, and volatility-related event sensors. Named entity recognition (NER) models were employed to detect company entities, policy institutions, and financial indicators as structured semantic sensor tags. Raw textual sensor streams underwent standardized sensor cleaning procedures, including deduplication, HTML signal stripping, language detection, and tokenization prior to semantic encoding. Extracted semantic sensing attributes include text length signals, sentiment polarity sensor scores, event category sensor labels, and high-dimensional semantic embedding vectors generated by multilingual pre-trained language sensor encoders. To ensure the reliability of these inputs, the performance of the keyword extraction and NER modules was validated against established NLP benchmarks and further refined through manual auditing of a stratified sample of the corpus, confirming the accuracy of the extracted structured tags in capturing meaningful financial signals. The resulting multilingual semantic sensing dataset contains approximately 850,000 English news sensing records, 620,000 Chinese news and disclosure sensing documents, and 2.1 million social media semantic sensing posts, forming a large-scale semantic sensor corpus exceeding 3.5 million sensing samples. All semantic sensor records were temporally mapped to corresponding trading days and minute-level sensing windows via timestamp calibration, enabling precise cross-sensor alignment with market behavioral sensing streams and providing a high-quality multimodal sensing foundation for subsequent cross-modal anomaly sensing model training. The reliability and consistency of this temporal mapping process were rigorously verified through randomized sampling audits and cross-validation with exchange matching-system logs, ensuring that the aligned dataset accurately reflects the chronological sequence of event dissemination and market reaction.

### 3.2. Data Preprocessing and Augmentation Strategy

Within the multilingual semantic–numerical collaborative sensing framework, data preprocessing and data augmentation are not merely engineering procedures for improving model stability, but constitute critical theoretical components for ensuring effective cross-modal collaborative modeling. Specifically, the high-frequency nature of modern electronic markets, characterized by rapid information diffusion and algorithmic execution, necessitates a preprocessing pipeline that can reconstruct market steady-states while simulating the temporal dynamics of shock propagation. Given the substantial discrepancies between numerical time-series data and textual semantic data in terms of statistical distributions, temporal scales, and noise structures, the absence of systematic preprocessing and structured enhancement may result in distribution shift, modality imbalance, or overfitting during model training. Accordingly, preprocessing and robust augmentation strategies are designed from two complementary perspectives, namely modality steady-state reconstruction and shock propagation simulation. Structured preprocessing and robustness enhancement are applied separately to the numerical and semantic modalities, while cross-modal perturbation mechanisms are introduced to strengthen adaptability to temporal misalignment scenarios.

On the numerical modality side, high-frequency financial time series commonly contain missing observations, abnormal spikes, and scale drift, which may arise from trading suspensions, matching delays, or extreme trading activities. Due to the high-frequency characteristics of financial markets, where risk signals often manifest within very short time horizons, the numerical sensor data are sampled at a base temporal resolution of one minute. This minute-level resolution is essential for preserving microstructure-level sensing granularity, allowing the framework to observe transient anomalies, high-velocity price discovery processes, and intraday liquidity fluctuations that are critical for forward-looking risk perception. Direct model input without correction may lead to gradient instability and misclassification of anomalies. Therefore, missing values are first reconstructed via interpolation. Let the original time series be denoted by $X_{t}\in R^{d}$. If an observation is missing at time *t*, local linear interpolation is employed for reconstruction as

(1) $${\tilde{X}}_{t}=X_{t_{1}}+\frac{t-t_{1}}{t_{2}-t_{1}}\left(X_{t_{2}}-X_{t_{1}}\right),$$

where $t_{1}$ and $t_{2}$ represent the nearest valid observation timestamps before and after the missing interval, respectively. This strategy preserves local trend continuity while avoiding additional structural assumptions. To mitigate the excessive influence of extreme spike values on parameter estimation, a sliding-window-based robust smoothing mechanism is introduced. Let the window size be *w*, then the smoothed sequence is expressed as

(2) $${\bar{X}}_{t}=\frac{1}{w}\sum_{i=0}^{w-1}{\tilde{X}}_{t-i}.$$

In practical implementation, median filtering can be combined to further enhance robustness against extreme values and suppress non-structural noise. To eliminate interference caused by scale heterogeneity across different indicators in attention weight allocation, standardization is performed. The mean μ and standard deviation σ are defined as

(3) $$\mu =\frac{1}{T}\sum_{t=1}^{T}{\bar{X}}_{t},\;\sigma =\sqrt{\frac{1}{T}\sum_{t=1}^{T}{\left({\bar{X}}_{t}-\mu \right)}^{2}},$$

and the normalized sequence is computed as

(4) $$X_{t}^{*}=\frac{{\bar{X}}_{t}-\mu }{\sigma }.$$

On this basis, multi-scale sliding time windows are constructed to enhance sensitivity to shocks occurring at different temporal resolutions. Let the window length be *L*, and the input sample is defined as

(5) $$X_{t}=\left\{X_{t-L+1}^{*},\ldots ,X_{t}^{*}\right\}.$$

By setting multiple scales $L_{1},L_{2},L_{3}$, multi-scale samples are constructed to enable simultaneous learning of short-term shocks and long-term trend structures, thereby improving anomaly recognition capability. On the semantic modality side, multilingual textual data exhibit expressive diversity and complex noise characteristics. The preprocessing objective is to construct a unified, robust, and alignable semantic representation space. Invalid characters, duplicate samples, and non-financial content are removed through text cleaning procedures, followed by financial terminology normalization to map semantically equivalent concepts across languages into unified expressions. For example, various multilingual expressions referring to “interest rate hikes” are mapped to a consistent event label to reduce semantic dispersion. Let the textual collection at time *t* be denoted by $T_{t}$, and the cleaned and standardized collection be represented as $T_{t}^{*}$.

A multilingual language model $f_{\mathrm{LLM}}$ is employed to encode both the standardized corpus and its augmented variants into a unified semantic embedding space. The selection of $f_{\mathrm{LLM}}$ is prioritized based on its robust cross-lingual semantic alignment capability, which ensures that textual data from disparate language spaces are mapped into a unified vector space where semantically similar events share proximal representations. This choice is particularly advantageous for financial terminology normalization, as it allows the model to effectively bridge linguistic variations and map heterogeneous expressions of the same financial concepts—such as various multilingual terms for “interest rate hikes”—into a consistent latent space, thereby enhancing the stability of the subsequent cross-modal alignment. Specifically, the semantic representations of the original and augmented textual samples are obtained as

(6) $$S_{t}=f_{\mathrm{LLM}}\left(T_{t}^{*}\right),\;\hat{S}t=f\mathrm{LLM}\left({\hat{T}}_{t}\right),$$

where $S_{t},{\hat{S}}_{t}\in R^{d_{s}}$ denote the embeddings of the cleaned corpus and the augmented corpus, respectively. Considering that financial anomaly events are typically low-frequency and sudden, reliance solely on original textual samples may lead to class imbalance. To alleviate this issue and enhance semantic diversity while preserving event consistency, data augmentation strategies are introduced to expand the textual distribution. Back-translation generates semantically preserved yet lexically varied samples by translating text into another language and subsequently back to the original language. Semantic synonym replacement substitutes keywords with semantically similar word vectors in the embedding space. Event template expansion further constructs structured samples based on predefined “subject–action–object” templates. The resulting augmented corpus $\hat{T}t$ shares the same encoder fLLM, ensuring that both original and augmented samples are aligned within a consistent semantic representation space. To enhance robustness against expressive perturbations, a contrastive loss between original and augmented representations is minimized as

(7) $$L_{\mathrm{contrast}}=-\log\frac{\exp(\mathrm{sim}\left(S_{t},{\hat{S}}_{t}\right)/\tau )}{\sum_{j}\exp\left(\mathrm{sim}\left(S_{t},S_{j}\right)/\tau \right)},$$

where sim(·) denotes cosine similarity and τ represents a temperature coefficient. At the cross-modal level, to simulate temporal misalignment between semantic shocks and market responses in real-world financial systems, a cross-modal synchronous perturbation augmentation mechanism is introduced. Let the semantic shock occur at time *t*, while the market response may emerge at t+δ, where δ denotes a dynamic time lag. To improve adaptability to varying δ, a random perturbation offset ϵ∼U(−Δ,Δ) is sampled during training to construct perturbed semantic samples as

(8) $${\tilde{S}}_{t}=S_{t+\epsilon }.$$

The numerical sample $X_{t}$ is kept unchanged, thereby simulating scenarios in which semantic signals lead or lag market responses. Through the incorporation of such perturbed samples during training, more robust cross-modal alignment strategies can be learned, reducing anomaly misclassification caused by temporal misalignment.

### 3.3. Problem Formulation

The objective of the proposed multilingual semantic–numerical collaborative sensing framework is to identify financial anomalies by modeling the joint distribution of heterogeneous sensor streams. Let the market behavior sensing space be represented by a numerical time-series sequence $X=\{x_{t-L+1},\ldots ,x_{t}\}$, where $x_{t}\in R^{d_{x}}$ denotes a multi-dimensional vector of price, volume, and order-book dynamics at time *t*, and *L* represents the sensing window length. Simultaneously, let the multilingual semantic sensing space be represented by a collection of unstructured textual records $T_{t}=\left\{\tau_{t,1},\ldots ,\tau_{t,k}\right\}$, where each τ represents a news article, disclosure document, or social media post captured within the same temporal horizon.

The anomaly sensing task is formulated as a binary classification problem. We aim to learn a non-linear mapping function $f:\left(X,T_{t}\right)\to y_{t}$, where $y_{t}\in \left\{0,1\right\}$ is the anomaly label. Here, $y_{t}=1$ indicates an anomalous state characterized by significant deviations in market behavior induced by exogenous semantic shocks, while $y_{t}=0$ represents a normal trading regime. The optimization objective is to minimize a joint loss function L, defined as

(9) $$L=L_{cls}\left({\hat{y}}_{t},y_{t}\right)+\lambda L_{align}\left(S_{t},{\hat{S}}_{t}\right),$$

where $L_{cls}$ is the binary cross-entropy loss for anomaly detection accuracy, $L_{align}$ is the contrastive alignment loss for cross-modal consistency, and λ is a balancing coefficient. Through this formulation, the framework seeks to find an optimal decision boundary in the high-dimensional latent space that maximizes the separability between anomalous and normal sensing states.

### 3.4. Proposed Method

#### 3.4.1. Overall

After standardized representations of the numerical sequences and multilingual texts have been obtained, an end-to-end multilingual semantic–numerical collaborative sensing Transformer framework is constructed from the model design perspective. The overall pipeline is sequentially connected and jointly optimized through five stages, namely semantic encoding, numerical encoding, cross-modal alignment, collaborative fusion, and anomaly decision. For the semantic input at time step *t*, the multilingual text set within the same temporal window is denoted by $T_{t}$. A multilingual large language model encoder $f_{\mathrm{LLM}}\left(\cdot \right)$ is employed to generate semantic embeddings $S_{t}=f_{\mathrm{LLM}}\left(T_{t}\right)$. A semantic noise-robust encoding module is then applied to incorporate confidence-aware weighting and contrastive regularization, such that the semantic representations remain stable under cross-lingual expression perturbations, yielding a denoised semantic sequence ${\left\{S_{t}\right\}}_{t=1}^{T}$. In parallel, on the numerical side, market behavior features within a time window of length *L* are organized as $X_{t}$ based on a sliding-window construction, and a temporal Transformer numerical encoder is applied to obtain a market dynamic latent representation $H_{t}={\mathrm{Trans}}_{\mathrm{num}}\left(X_{t}\right)$, thereby extracting temporal structures of behavioral signals such as price, volume, and order book dynamics under long-range dependency modeling. The cross-modal temporal alignment attention module is subsequently introduced to account for dynamic delays between semantic shocks and market responses. Specifically, $H_{t}$ is used as the query, while the semantic sequence $\left\{S_{\tau }\right\}$ provides keys and values, and a cross-attention operation yields an aligned semantic representation ${\tilde{S}}_{t}$. This mechanism enables adaptive selection of the most relevant semantic moments within a neighborhood time range to explain the current market state, explicitly mitigating misalignment between textual events and numerical fluctuations. After alignment, the semantic–numerical collaborative risk fusion module projects ${\tilde{S}}_{t}$ and $H_{t}$ into a unified latent space and generates a risk contribution weight $g_{t}$ through a gating mechanism, producing a fused risk representation $Z_{t}$ that models the coupling between semantic shock intensity and market fluctuation magnitude while enabling adaptive information allocation. Finally, $Z_{t}$ is fed into an anomaly decision head to output an anomaly probability $p_{t}=\sigma \left(\mathrm{MLP}\left(Z_{t}\right)\right)$. End-to-end training is conducted using supervised anomaly labels, jointly optimized with the contrastive regularization introduced in the semantic robust encoding stage, so that anomaly recognition accuracy and early warning capability are improved while semantic interpretability is preserved.

#### 3.4.2. Cross-Modal Temporal Alignment Attention Mechanism

The core of the cross-modal temporal alignment attention mechanism lies in overcoming the limitation of standard self-attention, which models dependencies only within a single modality, by dynamically coupling semantic and numerical sequences along the temporal dimension. In standard self-attention, queries, keys, and values are derived from the same input sequence, and the computation can be expressed as

(10) $$\mathrm{Attn}(X)=\mathrm{Softmax}\left(\frac{QK^{⊤}}{\sqrt{d}}\right)V,$$

where $Q=XW_{q}$, $K=XW_{k}$, and $V=XW_{v}$. This structure assumes that sequences are already aligned on the same temporal scale and is therefore inadequate for handling temporal misalignment between semantic shocks and market responses. In contrast, the proposed framework implements cross-modal interaction through a targeted retrieval process where the numerical dynamic representation acts as the query to search across the semantic sequence. This represents a structural improvement over standard Transformer blocks, as it replaces intra-modal dependency modeling with an explicit cross-modal mapping, thereby allowing the model to bridge the representational gap between market behaviors and textual events.

**Figure 1:**
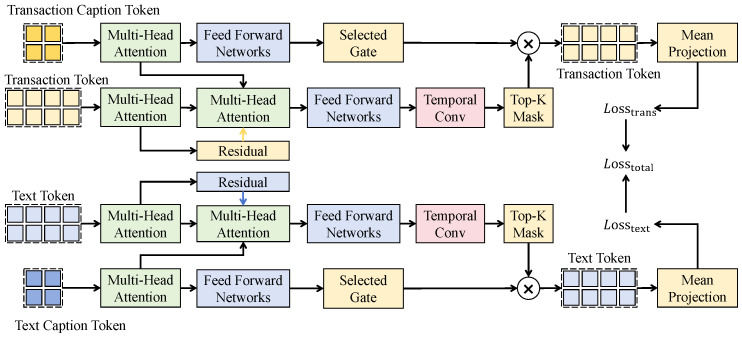
Illustration of the cross-modal temporal alignment attention mechanism.

As shown in Figure 1, the cross-modal temporal alignment attention mechanism adopts heterogeneous query and key–value sources. Let the numerical sequence representation be $H\in R^{T\times d_{h}}$ and the semantic sequence representation be $S\in R^{T\times d_{s}}$. Both modalities are first projected into a shared dimension *d* through linear mappings, yielding $Q=HW_{q}$, $K=SW_{k}$, and $V=SW_{v}$, enabling cross-modal attention to be computed as

(11) $$\mathrm{CMA}(H,S)=\mathrm{Softmax}\left(\frac{QK^{⊤}}{\sqrt{d}}\right)V.$$

The output encodes the contribution of the most relevant semantic moments under the current market state. In terms of network architecture, stacked cross-attention blocks are employed. Each block consists of a cross-modal attention sub-layer, residual connections, layer normalization, and a feed-forward network. The height corresponds to the temporal length *T*, the width corresponds to the shared latent dimension *d*, and the channel dimension remains consistent across layers to ensure stable residual propagation. Let the input to layer *l* be $Z^{\left(l-1\right)}$; then, the aligned representation is computed as

(12) $$Z^{\left(l\right)}=\mathrm{LayerNorm}\left(Z^{\left(l-1\right)}+\mathrm{CMA}\left(Z^{\left(l-1\right)},S\right)\right),$$

followed by a feed-forward transformation

(13) $$Z^{\left(l\right)}=\mathrm{LayerNorm}\left(Z^{\left(l\right)}+\mathrm{FFN}\left(Z^{\left(l\right)}\right)\right),$$

where FFN(·) consists of two linear transformations with a nonlinear activation. Unlike self-attention, the queries are consistently derived from the numerical modality, while keys and values are derived from the semantic modality, thereby enabling a mechanism of market-state-driven semantic retrieval rather than intra-modality dependency modeling.

To characterize temporal lag effects, a learnable time-offset kernel function g(τ) is introduced to locally reweight the semantic sequence, such that attention weights depend not only on feature similarity but also explicitly on temporal distance. Specifically, the introduction of this learnable time-offset kernel g(τ), which is not present in the standard Transformer architecture, serves as a specialized mechanism to explicitly resolve temporal misalignment between semantic shocks and market responses by modulating attention scores based on the relative time distance. Let the time offset be Δ=t−τ. The alignment weight is defined as

(14) $$\alpha_{t,\tau }=\frac{\exp\left(\frac{Q_{t}K_{\tau }^{⊤}}{\sqrt{d}}-\gamma \left|\Delta \right|\right)}{\sum_{j}\exp\left(\frac{Q_{t}K_{j}^{⊤}}{\sqrt{d}}-\gamma \left|t-j\right|\right)},$$

where γ denotes a learnable decay coefficient. If a semantic perturbation term is modeled as a zero-mean random variable $\epsilon_{\tau }$, then the output noise variance satisfies

(15) $$\mathrm{Var}\left(\sum_{\tau }\alpha_{t,\tau }\epsilon_{\tau }\right)=\sum_{\tau }\alpha_{t,\tau }^{2}\mathrm{Var}\left(\epsilon_{\tau }\right).$$

Since $\sum_{\tau }\alpha_{t,\tau }=1$ and $\alpha_{t,\tau }$ decays exponentially with increasing temporal distance, this variance is bounded above by the variance of the original noise term, thereby suppressing irrelevant long-range semantic perturbations. The mechanism therefore provides time-constrained noise contraction in theory and enables learning of an optimal alignment path between semantic shocks and market responses in practice, improving stability and forward-looking capability of anomaly detection under dynamic lag scenarios.

#### 3.4.3. Multilingual Semantic Noise-Robust Encoding Module

The multilingual semantic noise-robust encoding module is deployed in the semantic branch of the overall framework, aiming to construct a stable and alignable semantic representation space under cross-lingual expression variation and textual noise interference.

**Figure 2:**
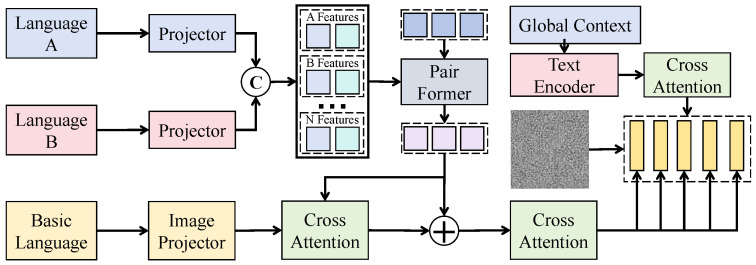
Illustration of the multilingual semantic noise-robust encoding module.

As shown in Figure 2, let the multilingual text set at time step *t* be encoded by a base language model into an initial semantic tensor $E_{t}\in R^{H\times W\times C}$, where *H* denotes the sequence-length dimension, *W* denotes the number of semantic subspace partitions, and *C* denotes the channel dimension. The tensor is then fed into a multi-layer robust semantic mapping network composed of stacked linear mappings and normalization layers, where each layer performs

(16) $$F^{\left(l\right)}=\phi \left(\mathrm{LN}\left(F^{\left(l-1\right)}W^{\left(l\right)}+b^{\left(l\right)}\right)\right),$$

with $F^{\left(0\right)}=E_{t}$. Here, $W^{\left(l\right)}\in R^{C\times C}$ is a learnable weight matrix, $b^{\left(l\right)}$ is a bias term, LN(·) denotes layer normalization, and ϕ(·) denotes a nonlinear activation. This mapping preserves the spatial dimensions H×W while reconstructing features along the channel dimension, thereby mitigating distribution drift induced by cross-lingual expression differences. To further characterize semantic confidence, a semantic consistency estimation function g(·) is introduced to produce a confidence weight tensor $C_{t}\in R^{H\times W\times 1}$, defined as

(17) $$C_{t}=\sigma \left({\mathrm{Conv}}_{1\times 1}\left(F^{\left(L\right)}\right)\right),$$

where σ(·) is the sigmoid function and ${\mathrm{Conv}}_{1\times 1}$ denotes a channel-wise compression mapping. The final denoised semantic representation is defined as

(18) $${\tilde{E}}_{t}=C_{t}⊙F^{\left(L\right)}.$$

To ensure that the denoising process is contractive in a statistical sense, it can be shown that when $C_{t}\in \left[0,1\right]$, the mapping ${\tilde{E}}_{t}=D\left(E_{t}\right)$ satisfies a norm contraction property:

(19) $$∥{\tilde{E}}_{t}{∥}_{2}\;=\;∥C_{t}⊙F^{\left(L\right)}{∥}_{2}\;\leq \;{∥F^{\left(L\right)}∥}_{2}.$$

Since $0\leq C_{t}\leq 1$, the element-wise product does not amplify vector magnitude, ensuring that noise components are suppressed in terms of energy. Considering an additive semantic perturbation ϵ in expectation, where the observed semantic representation is $E_{t}+\epsilon$, the denoised expectation becomes

(20) $$E\left[{\tilde{E}}_{t}\right]=E\left[C_{t}⊙\left(E_{t}+\epsilon \right)\right]=E\left[C_{t}⊙E_{t}\right]+E\left[C_{t}⊙\epsilon \right].$$

If the noise term satisfies E[ϵ]=0 and is weakly correlated with the confidence weights, the second term approaches zero, indicating that semantic principal components are preserved while random perturbations are suppressed in expectation, thereby improving representation stability. During training, a semantic consistency constraint is introduced to enforce angular alignment between original and augmented representations in embedding space. Let the augmented semantic tensor be denoted as ${\hat{E}}_{t}$. The consistency loss is defined as

(21) $$L_{\mathrm{align}}=1-\frac{\langle {\tilde{E}}_{t},{\tilde{\hat{E}}}_{t}\rangle }{∥{\tilde{E}}_{t}{∥}_{2}{∥{\tilde{\hat{E}}}_{t}∥}_{2}}.$$

This constraint regularizes the angle between the two representations on the unit hypersphere, thereby preserving semantic direction under cross-lingual and expression perturbations. Through the above design, the spatial dimension H×W is preserved while adaptive recalibration and compression are performed along the channel dimension, yielding a confidence-modulated semantic tensor ${\tilde{E}}_{t}$ that is subsequently fed into the cross-modal temporal alignment module. The advantages are twofold: noise energy is bounded through contractive mapping, and semantic principal directions are stabilized through consistency regularization, thereby reducing the impact of low-quality texts on anomaly decision boundaries and improving robustness and generalization in multilingual complex contexts.

#### 3.4.4. Semantic–Numerical Collaborative Risk Fusion Module

The semantic–numerical collaborative risk fusion module is positioned after cross-modal temporal alignment and achieves deep coupling of the two modalities within a unified risk space through gating modulation and expert-mixture-style interaction.

**Figure 3:**
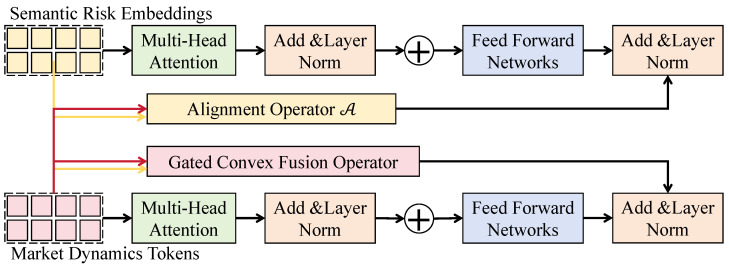
Illustration of the semantic–numerical collaborative risk fusion module.

As shown in Figure 3, let the aligned semantic representation be ${\tilde{S}}_{t}\in R^{H_{s}\times C_{s}}$ and the numerical encoder output be $H_{t}\in R^{H_{n}\times C_{n}}$. Both are first projected to a shared channel dimension *C* via linear layers:

(22) $$U_{t}={\tilde{S}}_{t}W_{s},\;V_{t}=H_{t}W_{n},$$

where $W_{s}\in R^{C_{s}\times C}$ and $W_{n}\in R^{C_{n}\times C}$. A cross-modal collaborative mapping tensor $M_{t}\in R^{H\times C}$ is then constructed as

(23) $$M_{t}=\mathrm{LN}\left(U_{t}+V_{t}+\Psi \left(U_{t}⊙V_{t}\right)\right),$$

where ⊙ denotes element-wise multiplication, Ψ(·) denotes a channel compression mapping, and LN denotes layer normalization. This structure preserves the spatial length *H* while capturing coupling between semantic shock intensity and numerical fluctuation magnitude through multiplicative interactions. To improve distributional adaptability of risk representations, an adaptive gating weight tensor $G_{t}\in R^{H\times C}$ is computed as

(24) $$G_{t}=\sigma \left(M_{t}W_{g}\right),$$

where $W_{g}\in R^{C\times C}$ and σ(·) is the sigmoid function. The fused representation is defined as

(25) $$Z_{t}=G_{t}⊙U_{t}+\left(1-G_{t}\right)⊙V_{t}.$$

This expression is functionally equivalent to a convex combination of semantic and numerical subspaces. Since $G_{t}\in \left[0,1\right]$, it can be shown that the fusion operator is a bounded linear operator with the spectral norm satisfying

(26) $${∥Z_{t}∥}_{2}\;\leq \;{∥U_{t}∥}_{2}+\;{∥V_{t}∥}_{2},$$

thereby ensuring that risk representations remain within a numerically stable range. Furthermore, considering the Lipschitz continuity of a risk response function $R(Z_{t})$ under input perturbations, if the semantic and numerical encoding mappings are Lipschitz continuous, their composition preserves Lipschitz properties, yielding stable responses to cross-modal perturbations. When jointly employed with the cross-modal temporal alignment module, temporally matched ${\tilde{S}}_{t}$ is first obtained through alignment attention, and a unified risk tensor is subsequently constructed through the collaborative fusion operator. The combined process can be expressed as a composite mapping:

(27) $$Z_{t}=F\left(A\left(S_{1:T},H_{1:T}\right)\right),$$

where A denotes the temporal alignment operator and F denotes the collaborative fusion operator. Since A performs time-domain rearrangement while F performs feature-domain coupling, the two operations are functionally complementary and jointly integrate risk information across temporal and representational dimensions. Architecturally, the fusion module is embedded between dual-branch Transformer blocks with residual connections and feed-forward networks forming closed-loop updates. The channel dimension *C* is preserved across layers, and the spatial length *H* matches the input time-step length, ensuring alignment of cross-modal information at each temporal position. In financial anomaly detection, this design adaptively adjusts the contribution of semantic shocks and market behaviors under varying contexts, strengthening the semantic channel under policy-driven or event-driven shocks and strengthening the numerical channel under technically driven fluctuations, thereby optimizing adaptive decision boundaries within a unified risk space and improving anomaly detection accuracy and cross-market generalization.

## 4. Results and Discussion

### 4.1. Experimental Configuration

#### 4.1.1. Hardware and Software Platform

The experiments were conducted on a high-performance computing platform. The hardware environment was built upon a dual-processor server architecture equipped with Intel Xeon Gold series multi-core CPUs operating at frequencies above 2.6 GHz, and 128 GB DDR4 memory to support parallel loading and in-memory computation of high-frequency financial data and large-scale textual corpora. The graphics processing unit consisted of an NVIDIA A100 with 80 GB memory, which was utilized for multilingual large language model semantic encoding and accelerated training of the cross-modal Transformer framework. Local storage was configured with a 2 TB NVMe solid-state drive to ensure efficient high-frequency data read–write operations, complemented by a 10 Gbps network interface to support real-time synchronization of multi-market data and scalability to distributed training environments. The overall hardware configuration satisfied the computational requirements of large-scale multimodal training and high-frequency temporal inference, while ensuring the reproducibility and stability of the experimental results.

The software environment was established on Ubuntu 22.04 LTS. The deep learning framework was implemented using PyTorch 2.1, integrated with CUDA 12.1 and the cuDNN acceleration library to enable GPU-parallel computation. The multilingual semantic encoding module was explicitly implemented based on the HuggingFace Transformers library, with specific pre-trained multilingual language model weights such as mBERT and XLM−R loaded for robust semantic embedding generation. Data preprocessing and analysis were executed within a Python 3.10 environment using scientific computing libraries including NumPy 1.24, Pandas 1.5, and Scikit-learn 1.2 for feature construction and metric computation. Experiment management and visualization were conducted using TensorBoard 2.12 and Weights and Biases 0.15 for logging and monitoring. Mixed-precision training strategies were adopted to enhance computational efficiency and reduce GPU memory consumption. The overall software configuration ensured efficient and scalable training of the cross-modal anomaly detection framework.

Regarding hyperparameter configuration, the dataset was chronologically partitioned into training, validation, and testing sets with proportions of 70%, 15%, and 15%, respectively, to preserve causal consistency in time-series modeling. In addition, five-fold cross-validation was introduced during training, where stratified splits were applied to mitigate class imbalance and assess model stability. The numerical modality time window length L was set to 60 time steps. This duration represents one hour of trading at our base one-minute resolution, providing an optimal sensing horizon to capture intraday microstructure dynamics while maintaining computational efficiency. To further ensure robustness, additional multi-scale windows of 30 and 120 steps were introduced to enhance sensitivity to transient short-term shocks and sustained long-term trends, respectively. The hidden dimension dh was set to 256, and the semantic embedding dimension ds was set to 768. The Transformer encoder consisted of 4 layers with 8 attention heads, and a dropout ratio p of 0.2 was applied to prevent overfitting. The optimizer adopted was AdamW, with an initial learning rate alpha = $1\times 10^{-4}$ and cosine annealing scheduling applied for learning rate decay. The batch size B was set to 64, and the number of training epochs was fixed at 50. An early stopping strategy was employed, whereby training was terminated if the validation area under curve (AUC) did not improve for 10 consecutive epochs. The contrastive learning temperature parameter tau was set to 0.07, and the weight decay coefficient lambda was set to $1\times 10^{-5}$. These hyperparameters were determined through a combination of grid search and cross-validation to ensure optimal performance and generalization capability in the multilingual semantic–numerical collaborative anomaly detection task.

#### 4.1.2. Baseline Models and Evaluation Metrics

To comprehensively evaluate the effectiveness of the proposed multilingual semantic–numerical collaborative sensing framework, several representative baseline models are selected for comparison. ARIMA-GARCH [31], as a classical statistical modeling approach, captures autocorrelation structures and volatility clustering characteristics in time series and provides strong theoretical interpretability and stability in financial risk modeling. Isolation Forest [32] identifies anomalies based on feature-space isolation mechanisms and offers the advantage of detecting abnormal samples without requiring labeled data, making it suitable for complex high-dimensional scenarios. LSTM-AE [33] integrates recurrent neural networks with autoencoding structures to effectively capture long-term dependencies and learn normal behavioral patterns, enabling anomaly detection through reconstruction error modeling. TCN [34] models long-range temporal dependencies via multi-scale dilated convolutions and exhibits high parallel computation efficiency and stable gradient propagation. The numerical-only Transformer [35] leverages self-attention mechanisms to model global dependencies and demonstrates strong representational capability in long-sequence dynamic modeling. The text-only model [36] employs a multilingual large language model for semantic encoding, effectively capturing cross-lingual sentiment and event structures with strong semantic generalization ability. The late fusion model [37] achieves cross-modal integration through feature concatenation and multilayer perceptrons, offering structural simplicity and extensibility. The simple cross-attention fusion model [38] introduces cross-attention mechanisms to model correlations between semantic and numerical representations, enabling deeper interaction at the fusion level. To reflect the state-of-the-art (SOTA), we further incorporate MLLM-Anomaly [24], which utilizes multimodal large language models for zero-shot reasoning and risk identification, and the Contrastive Multimodal Transformer (CMT) [15], which employs contrastive learning objectives to align heterogeneous representations in a shared latent space.

Precision, recall, F1-score, Area Under the Curve (AUC), Matthews Correlation Coefficient (MCC), and early warning time (EWT) were adopted as comprehensive evaluation metrics. Precision measures the proportion of correctly identified anomalies among all predicted anomalies. Recall evaluates the proportion of correctly detected anomalies among all true anomalies. F1-score provides a harmonic balance between precision and recall. AUC characterizes the overall discriminative capability of the model under varying decision thresholds. MCC provides a balanced measure that can be used even if the classes are of very different sizes, offering a robust assessment of model performance under the typical class imbalance of financial anomaly detection. EWT assesses the temporal advantage of detection relative to the actual occurrence of anomalies. Let the confusion matrix consist of true positives TP, false positives FP, false negatives FN, and true negatives TN. The evaluation metrics are defined as follows:

(28) $$Precision=\frac{TP}{TP+FP},$$

(29) $$Recall=\frac{TP}{TP+FN},$$

(30) $$F1=\frac{2\times Precision\times Recall}{Precision+Recall},$$

(31) $$AUC=\int_{0}^{1}TPR\left(FPR\right)\;d\left(FPR\right),$$

(32) $$MCC=\frac{TP\times TN-FP\times FN}{\sqrt{\left(TP+FP)(TP+FN)(TN+FP)(TN+FN\right)}},$$

(33) $$EWT=T_{\mathrm{anomaly}}-T_{\mathrm{detect}}.$$

Here, TP denotes the number of samples that are true anomalies and correctly detected; FP denotes the number of normal samples incorrectly classified as anomalies; FN denotes the number of anomaly samples that were not detected; and TN denotes the number of normal samples correctly identified. The true positive rate is defined as $TPR=\frac{TP}{TP+FN}$, and the false positive rate is defined as $FPR=\frac{FP}{FP+TN}$. The variable $T_{\mathrm{anomaly}}$ represents the actual occurrence time of an anomaly, and $T_{\mathrm{detect}}$ denotes the time at which the model first generates an anomaly signal.

### 4.2. Anomaly Detection Performance Comparison

The purpose of this experiment is to systematically compare the performance differences among various modeling paradigms in the multi-market anomaly detection task, thereby validating the advantages of the semantic–numerical collaborative sensing framework in terms of accuracy, stability, and forward-looking capability. The comparison encompasses traditional statistical models, unsupervised anomaly detection approaches, deep temporal models, single-modality semantic models, and different cross-modal fusion strategies, enabling comprehensive evaluation from the perspectives of numerical behavior modeling, semantic event modeling, and cross-modal collaborative modeling.

**Figure 4:**
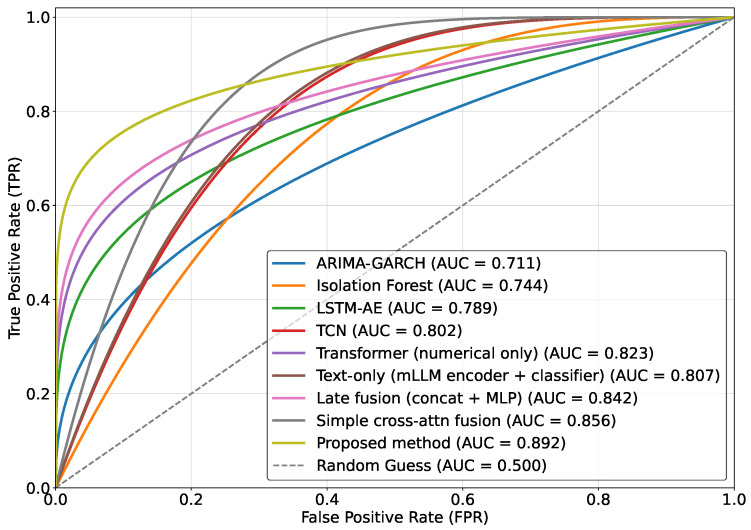
ROC curve for anomaly detection performance comparison across multiple models. The proposed MSNCformer achieves a superior AUC of 0.892, significantly outperforming traditional statistical models and deep temporal baselines. This performance gain is primarily attributed to the synergy of cross-modal temporal alignment and noise-robust semantic encoding, which effectively enlarges the decision margin between anomalous and normal market states.

As shown in Table 2 and Figure 4, ARIMA-GARCH exhibits overall inferior performance across all metrics, particularly in recall and EWT, indicating that statistical models based on linear stationarity assumptions and conditional heteroscedastic structures are insufficient to capture nonlinear structural changes induced by abrupt semantic shocks. Isolation Forest demonstrates moderate improvement over statistical models; however, due to its reliance on feature-space isolation without explicit temporal dynamic modeling, performance remains limited under complex continuous fluctuation scenarios. LSTM-AE and TCN achieve noticeable improvements in F1-score and AUC, suggesting that nonlinear sequence modeling and multi-scale convolutional structures are more effective in capturing price dynamics, yet they remain confined to historical numerical behavior modeling. The numerical-only Transformer further enhances overall performance, reflecting the superiority of self-attention mechanisms in modeling long-range dependencies; nevertheless, its EWT remains substantially lower than that of fusion-based approaches, indicating that reliance solely on price structures restricts early semantic-driven anomaly detection.

The text-only model achieves relatively higher recall and EWT compared to certain numerical models, highlighting the forward-looking nature of semantic information. However, its precision remains limited due to the absence of market-behavior validation. Late fusion improves all metrics over single-modality models through simple concatenation, yet the absence of explicit temporal alignment and coupling modeling constrains performance gains. The simple cross-attention fusion model further improves results, demonstrating the importance of cross-modal interaction modeling in anomaly detection. The advanced MLLM-Anomaly model exhibits strong reasoning capabilities and high EWT, yet its precision is affected by the lack of fine-grained temporal alignment with microstructure signals. CMT achieves competitive stability by leveraging contrastive learning for representation alignment, but it still falls short of the proposed method’s performance.

Furthermore, the introduction of the Matthews Correlation Coefficient (MCC) provides a more robust assessment of model performance under the typical class imbalance of financial anomaly detection. The proposed method achieves an MCC of 0.796, significantly outperforming all baselines, which confirms its superior capability in minimizing both false positives and false negatives in skewed distributions. Regarding computational efficiency, while numerical-only models maintain lower latency due to their simpler structures, the proposed method achieves an inference latency of 19.36 ms per sample. Despite the inclusion of complex cross-modal alignment and multilingual encoding, this latency remains well within the requirements for real-time financial monitoring, striking an optimal balance between sensing depth and operational efficiency.

The proposed method achieves the best performance across all metrics, with particularly significant advantages in AUC and EWT. This success is fundamentally attributed to the synergy of the three core modules: the temporal alignment module corrects the time bias between semantic events and market responses, the denoising module stabilizes the semantic representations against cross-lingual perturbations, and the gated fusion module effectively enhances feature coupling across modalities. By operating in concert, this architecture constructs a structurally constrained risk representation in the latent feature space, which serves to enlarge the decision margin for anomaly identification and improve overall discriminative power. From a mathematical perspective, the model reconstructs semantic–market mappings in the temporal domain through learnable alignment mechanisms and forms structurally constrained risk representations in feature space via gated collaborative operators. This design enhances cross-modal coupling strength while preserving representation stability, thereby enlarging the decision margin during boundary learning. Essentially, a more expressive and structurally constrained risk function is constructed in high-dimensional latent space, enabling clearer separability between anomalous and normal samples under joint semantic–behavioral distributions, which results in simultaneous improvements in precision, recall, and early warning capability.

### 4.3. Cross-Market Generalization Experiment

The cross-market generalization experiment is designed to evaluate model robustness and generalization under distribution shift conditions, specifically whether a model trained in one market environment can maintain high anomaly detection performance in another market characterized by different institutional structures, trading mechanisms, and investor behaviors. Bidirectional experiments, including training in the U.S. market and testing in the Chinese market, as well as the reverse configuration, provide a comprehensive assessment of adaptability to statistical distribution and semantic environment variations across markets.

**Figure 5:**
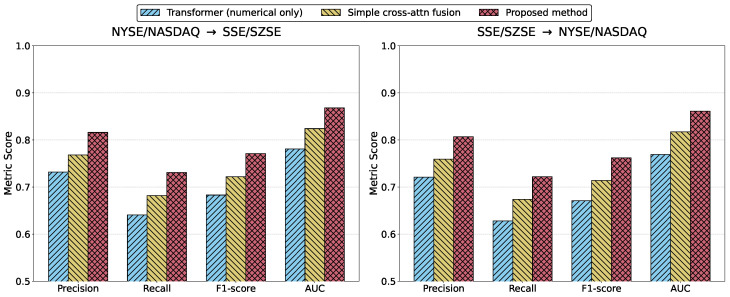
Bar chart of cross-market anomaly detection performance comparison under bidirectional transfer settings (U.S. to Chinese markets and vice-versa). The proposed method maintains the highest scores across precision, recall, and AUC in both directions, demonstrating that the semantic–numerical collaborative risk space effectively captures cross-market risk invariants and mitigates the performance degradation typically caused by numerical distribution shifts.

As shown in Table 3 and Figure 5, the numerical-only Transformer exhibits noticeable performance degradation under cross-market transfer, with lower precision, recall, and AUC compared to fusion-based models, and reduced EWT, indicating strong dependence on source-market price dynamics and sensitivity to distribution shifts. The simple cross-attention fusion model demonstrates consistent improvement in both transfer directions, suggesting that semantic information mitigates overfitting to numerical patterns. However, its recall and AUC remain inferior to those of the proposed method, reflecting limited depth in cross-modal modeling. The proposed method maintains the highest precision, recall, and AUC in both directions, with significant advantages in EWT, indicating stable forward-looking risk perception across heterogeneous market environments. From a theoretical standpoint, numerical-only models effectively learn market-specific autocorrelation structures and volatility patterns, resulting in latent representations tightly aligned with source-market statistics. Under distribution shift, learned decision boundaries fail to generalize effectively. Simple cross-attention fusion enhances representational richness through semantic integration, yet lacks structured temporal alignment and risk coupling, resembling shallow linear combinations that are insufficient for handling inter-market heterogeneity. In contrast, the proposed framework constructs a semantic–numerical collaborative risk space that incorporates cross-modal constraints, reducing reliance on market-specific numerical distributions and integrating semantic shock structures with behavioral dynamics. From a functional mapping perspective, temporal alignment and gated fusion mechanisms abstract shared cross-market risk patterns, enabling improved decision stability and early warning performance under cross-market transfer.

### 4.4. Module Ablation Study

The ablation study is designed to evaluate the independent contributions and collaborative effects of each core module by systematically removing key components and analyzing changes in anomaly detection accuracy and early warning capability. This analysis reveals the functional roles of individual modules in the overall risk perception process.

**Figure 6:**
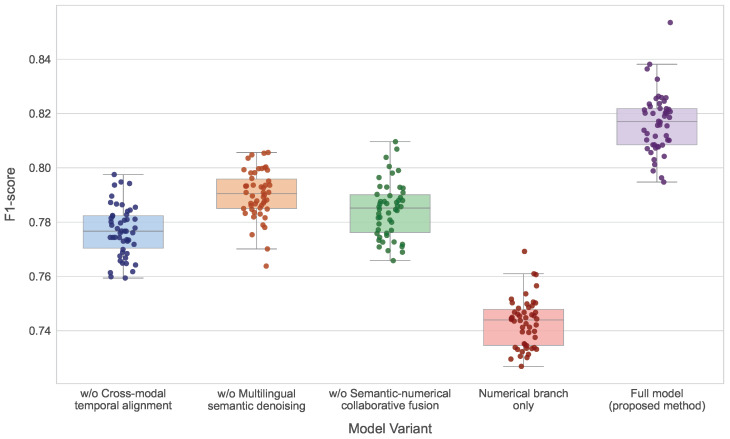
Box plot of F1-score distribution in module ablation experiments. The results highlight the independent and synergistic contributions of each core component. The full model consistently achieves the highest median F1-score, confirming that integrating temporal alignment, semantic denoising, and collaborative fusion is essential for achieving optimal sensing accuracy and robust early warning capability.

As shown in Table 4 and Figure 6, the numerical-only variant performs substantially worse than the full model across all metrics, particularly in AUC and EWT, demonstrating that market behavior sequences alone are insufficient to capture early anomaly signals. Removing the cross-modal temporal alignment module leads to declines in precision and recall, with EWT reduced from 8.9 to 6.4 min, indicating that temporal alignment plays a critical role in capturing semantic-leading and market-lagging structures. Eliminating the multilingual semantic denoising module results in noticeable degradation compared to the full model, highlighting the importance of stabilizing semantic representations and sharpening decision boundaries. Removing the semantic–numerical collaborative fusion module also reduces performance, suggesting that shallow combination strategies cannot adequately model the coupling between semantic shocks and market volatility. Theoretically, each module operates in distinct domains: temporal alignment corrects mismatches in time, semantic denoising stabilizes representation in semantic space, and collaborative fusion enhances feature coupling in latent space. Without temporal alignment, implicit synchronous assumptions introduce systematic bias in functional mappings. Without semantic denoising, representation noise perturbs optimization trajectories, degrading stability. Without collaborative fusion, decision functions rely on additive rather than interaction-based structures, reducing separability in high-dimensional space. The integrated model simultaneously achieves temporal correction, semantic stabilization, and risk coupling enhancement, resulting in optimal performance in accuracy, recall, and early warning capability.

### 4.5. Ablation Study on Multilingual Semantic Impact

The primary objective of this ablation experiment is to quantitatively evaluate the specific contribution of multilingual semantic modeling to anomaly detection performance and to verify whether the integration of cross-lingual information streams provides a significant advantage over strong monolingual baselines. By isolating the semantic inputs into English-only, Chinese-only, and the proposed multilingual configurations while keeping the numerical sensor streams and the cross-modal alignment architecture constant, we aim to observe how linguistic diversity affects the model’s ability to perceive global risk spillovers and information asymmetry. This experiment provides empirical evidence for the necessity of a multilingual sensing approach in the context of interconnected global capital markets.

As shown in Table 5, the experimental results demonstrate a clear performance hierarchy across the different linguistic configurations. The English-only version of the model achieves a moderate F1-score and Early Warning Time (EWT), primarily capturing global macroeconomic shifts and international industry shocks, while the Chinese-only version shows consistent precision in local market events but limited coverage of external systemic risks. In contrast, the proposed multilingual collaborative sensing framework achieves the highest performance across all metrics, with an F1-score improvement of approximately 5.2% over the English-only baseline and 6.8% over the Chinese-only baseline. Notably, the EWT of the multilingual model reaches 8.9 min, which is substantially higher than the 6.4 min and 5.9 min recorded for the monolingual variants, indicating that the simultaneous perception of cross-lingual information leads to a more rapid identification of emerging anomalies. The analysis of these results indicates that multilingual modeling significantly improves detection capability by mitigating information asymmetry and capturing early leads in international information propagation. Specifically, the framework is able to identify instances where news originating in one language space serves as a precursor to market volatility in another region, a dynamic that monolingual baselines inherently fail to perceive. From a representational perspective, the multilingual language model maps heterogeneous linguistic expressions into a unified semantic space, allowing the temporal alignment module to extract more comprehensive risk signals from a diverse information set. This confirms that the integration of multilingual semantics is not merely an expansion of the data volume but a structural enhancement that enables the model to perceive cross-market risk transmission paths more effectively than any single-modality or monolingual approach.

### 4.6. Parameter Sensitivity Analysis

The objective of this parameter sensitivity experiment is to quantitatively evaluate how the learnable temporal decay coefficient γ in the alignment kernel g(τ) influences the model’s ability to balance historical semantic context with instantaneous market shocks. As γ dictates the exponential decay rate of attention weights relative to temporal distance, this analysis aims to identify the optimal sensing horizon that maximizes anomaly detection accuracy while maintaining early warning advantages. By varying the initial values of γ and observing the resulting impact on detection performance and early warning time, we seek to characterize the trade-off between capturing long-range information propagation and suppressing irrelevant semantic noise.

As illustrated in Table 6, the model performance exhibits significant sensitivity to the magnitude of the decay coefficient. When γ is set to a very small value, such as 0.001, the model maintains a high Early Warning Time (EWT) of 9.4 min but suffers from a degradation in F1-score and Precision, as the broad attention span introduces excessive noise from distant and potentially irrelevant semantic records. Conversely, when γ is increased to 5.0, the sensing window becomes overly restrictive, forcing the model to focus only on the most recent semantic signals; while this suppresses noise, it severely diminishes the EWT to 3.4 min and reduces the F1-score by failing to capture the leading indicators of market volatility. The optimal performance is achieved at γ=0.1, where the framework strikes an effective balance between preserving forward-looking semantic leads and maintaining structural noise contraction.

### 4.7. Case Study

To further evaluate the practical utility and interpretability of the proposed MSNCformer, we conduct a qualitative analysis of representative sensing scenarios, focusing on both successful early warnings and challenging edge cases.

The best-performing cases typically involve “semantic-leading and market-lagging” structures, where an exogenous shock originates in the global information space before being reflected in price dynamics. For instance, during a major central bank policy announcement, our framework identified shifts in policy tone from English-language semantic sensors approximately 8.9 min before substantial price fluctuations occurred in Asian markets. By visualizing the attention maps of the cross-modal temporal alignment mechanism, we observed that the model assigned high weights to specific international news headers, effectively bridging the time gap between initial reporting and subsequent algorithmic trading responses. This early warning capability is attributed to the framework’s ability to identify instances where news in one language space serves as a precursor to volatility in another region.

Conversely, the most challenging scenarios involve high-frequency semantic noise or “false positive” signals where sudden textual shocks do not result in substantive market breakdowns. In one analyzed edge case, social media rumors and emotional discussions regarding a potential corporate restructuring triggered a spike in the semantic sensing signal. However, the numerical sensors, specifically order book depth and trading volume dynamics, remained relatively stable. In this instance, the semantic–numerical collaborative risk fusion module utilized its gated mechanism to adaptively decrease the contribution weight of the unreliable semantic channel, thereby preventing a persistent anomaly alert. Such cases highlight the importance of the multilingual semantic denoising module in filtering low-credibility texts to maintain representation stability under complex linguistic contexts. By analyzing these successes and failures, we confirm that the integration of temporal alignment and gated fusion is essential for distinguishing transient sentiment-driven fluctuations from substantive financial risk signals.

### 4.8. Discussion

The proposed multilingual semantic–numerical collaborative sensing framework demonstrates clear practical value in real-world financial scenarios. In increasingly interconnected global capital markets, macroeconomic policy releases, geopolitical events, public emergencies, and major corporate disclosures propagate across multiple languages and are rapidly amplified by algorithmic trading systems. For instance, when a central bank announces interest rate decisions or forward guidance, English-language media typically report first, followed by reinterpretations in Chinese and other languages, while price reactions may lag behind semantic diffusion. The proposed model identifies shifts in policy tone at the semantic level and determines potential market impact through cross-modal temporal alignment, enabling risk alerts before substantial price fluctuations occur. Such capability is directly applicable to brokerage risk management departments, quantitative trading teams, and exchange-level supervisory systems for dynamic position adjustment, risk limit setting, and trigger-based monitoring.

The robustness of the framework is further evidenced by its generalization capability during unseen time periods and extreme crisis events. Unlike traditional models that may overfit to specific market regimes, the proposed architecture captures structural risk invariants—universal patterns of how information shocks translate into price volatility. During historical intervals of high market turmoil, the model maintains high detection accuracy by identifying high-velocity information cascades that precede systemic failures, proving its reliability as a forward-looking sensing tool even in environments characterized by unprecedented volatility.

At the corporate level, announcements regarding mergers, restructurings, or earnings warnings may be expressed differently across languages, and social media discussions may contain emotional noise. The semantic denoising module filters low-credibility texts and extracts stable event representations, which are subsequently coupled with order book depth and trading volume dynamics to distinguish transient sentiment-driven fluctuations from substantive risk signals. Moreover, the practical applicability of the framework is significantly enhanced by its inherent interpretability. By visualizing the attention maps of the cross-modal temporal alignment mechanism, risk managers can trace the specific semantic triggers—such as a particular policy announcement or a corporate disclosure—that contributed to a specific anomaly alert. This feature attribution capability allows for a transparent audit trail, transforming the model from a black-box predictor into a diagnostic tool that reveals the causal links between multilingual information propagation and market behavioral responses.

In cross-market contexts, such as U.S. technology earnings influencing supply-chain-related stocks in Asian markets, the model maintains strong detection capability even when trained in a different market, indicating that learned risk representations capture shared semantic-driven patterns rather than relying solely on market-specific statistical features. This characteristic is valuable for cross-border asset allocation, global macro hedging strategies, and multi-market regulatory coordination, contributing to the development of more intelligent and forward-looking financial risk monitoring systems.

### 4.9. Limitation and Future Work

Although the multilingual semantic–numerical collaborative sensing framework demonstrates stable performance improvements across multiple markets, several directions warrant further investigation. The quality of semantic information heavily depends on publicly available textual sources and media reporting structures. In markets with incomplete disclosure or imbalanced language coverage, semantic signals may exhibit representational bias, potentially affecting comprehensive risk perception. In addition, the current modeling framework operates primarily at minute-level temporal resolution; validation under second-level high-frequency trading environments or longer-term macroeconomic evolution scenarios remains limited. While cross-market experiments indicate certain generalization capability, institutional and regulatory differences across countries may introduce more complex distribution shifts that require broader validation across additional markets and asset classes. Future research may incorporate finer-grained multi-source data, such as implied volatility, capital flow information, and supply chain network structures, and explore online learning and adaptive updating mechanisms to enhance sustained adaptability in dynamic financial environments.

## 5. Conclusions

Under the background of highly interconnected global capital markets and rapid cross-lingual information dissemination, traditional anomaly detection methods that rely solely on numerical time series have become insufficient for forward-looking risk identification. To address this practical challenge, a multilingual semantic–numerical collaborative sensing Transformer framework is constructed, forming an end-to-end modeling pipeline that integrates semantic encoding, cross-modal temporal alignment, and collaborative risk fusion, thereby enabling unified modeling of semantic shocks and dynamic market behaviors. At the methodological level, a cross-modal temporal alignment attention mechanism is introduced to model the dynamic propagation structure characterized by semantic leading and market lagging effects. A multilingual semantic noise-robust encoding module is incorporated to enhance the stability of cross-lingual representations. In addition, a semantic–numerical collaborative risk fusion module is designed to adaptively allocate risk contribution weights within a unified latent space, strengthening anomaly discrimination capability from the perspectives of temporal dynamics, semantic representation, and feature coupling. Experimental results validate the effectiveness of the proposed framework. In multi-market anomaly detection comparisons, the proposed method achieves the best performance across all evaluation metrics, including precision, recall, F1-score, and AUC. Specifically, precision reaches 0.852, recall reaches 0.781, F1-score achieves 0.815, and AUC improves to 0.892, while early warning time is significantly extended compared to various baseline models. In cross-market generalization experiments, high accuracy and stability are maintained under bidirectional transfer settings, with AUC consistently exceeding 0.86, demonstrating strong structural generalization capability. Module ablation studies further confirm that each core component contributes both independently and synergistically to overall performance improvement. Overall, a joint semantic–behavioral risk function is constructed at the theoretical level, and more forward-looking and stable financial anomaly detection is achieved in practice, providing a scalable technical framework for intelligent multi-market risk monitoring.

## Figures and Tables

**Table 1:** Multimodal Artificial Intelligence-Driven Financial Sensing Dataset Statistics.

Data Type	Sensing Modality	Data Source	Data Volume
High-frequency price streams	Market behavior sensor	NYSE (New York, NY, USA), NASDAQ (New York, NY, USA), SSE (Shanghai, China), SZSE (Shenzhen, China)	$4.5\times 10^{8}$
Order book depth streams	Microstructure sensor	Exchange real-time APIs (NYSE: New York, NY, USA; NASDAQ: New York, NY, USA; SSE: Shanghai, China; SZSE: Shenzhen, China)	$1.2\times 10^{8}$
English financial news	Semantic text sensor	Refinitiv (London, UK), Bloomberg (New York, NY, USA)	850,000
Chinese news and disclosures	Semantic text sensor	CNINFO (Shenzhen, China), Wind (Shanghai, China)	620,000
Social media financial posts	Public sentiment sensor	Twitter (San Francisco, CA, USA), financial forums (multiple platforms)	2,100,000
Total textual sensing data	Multilingual semantic sensor	Aggregated multi-source platforms	3,570,000

**Table 2:** Performance comparison of different baseline models on multi-market anomaly detection. Results are presented as mean ± standard deviation across five-fold cross-validation. The asterisk (*) denotes that the performance of the proposed method is statistically significant compared to the best baseline (*p* < 0.05) based on a paired *t*-test.

Method	Precision	Recall	F1-Score	AUC	MCC	EWT (min)	Latency (ms)
ARIMA-GARCH [31]	0.672 ± 0.012	0.541 ± 0.015	0.599 ± 0.011	0.711 ± 0.009	0.452 ± 0.014	2.6 ± 0.4	0.54 ± 0.02
Isolation Forest [32]	0.701 ± 0.010	0.603 ± 0.012	0.648 ± 0.009	0.744 ± 0.008	0.518 ± 0.011	3.1 ± 0.3	0.82 ± 0.03
LSTM-AE [33]	0.742 ± 0.008	0.661 ± 0.010	0.699 ± 0.007	0.789 ± 0.006	0.584 ± 0.009	3.8 ± 0.3	2.15 ± 0.08
TCN [34]	0.758 ± 0.009	0.684 ± 0.009	0.719 ± 0.008	0.802 ± 0.007	0.613 ± 0.010	4.2 ± 0.2	1.42 ± 0.05
Transformer (numerical only) [35]	0.781 ± 0.007	0.706 ± 0.008	0.742 ± 0.006	0.823 ± 0.005	0.655 ± 0.008	4.7 ± 0.2	3.28 ± 0.11
Text-only (mLLM encoder + classifier) [36]	0.736 ± 0.011	0.691 ± 0.013	0.713 ± 0.010	0.807 ± 0.008	0.628 ± 0.012	5.4 ± 0.5	15.42 ± 0.45
Late fusion (concat + MLP) [37]	0.803 ± 0.006	0.728 ± 0.007	0.764 ± 0.005	0.842 ± 0.004	0.681 ± 0.007	6.1 ± 0.3	16.18 ± 0.52
Simple cross-attn fusion [38]	0.818 ± 0.006	0.742 ± 0.007	0.778 ± 0.005	0.856 ± 0.005	0.709 ± 0.007	0.68 ± 0.3	18.45 ± 0.58
MLLM-Anomaly [24]	0.829 ± 0.005	0.755 ± 0.006	0.790 ± 0.005	0.865 ± 0.004	0.732 ± 0.006	7.2 ± 0.4	45.61 ± 1.25
CMT [15]	0.838 ± 0.005	0.766 ± 0.006	0.800 ± 0.004	0.877 ± 0.004	0.748 ± 0.006	7.8 ± 0.4	20.14 ± 0.62
Proposed method	0.852 ± 0.005 *	0.781 ± 0.006 *	0.815 ± 0.005 *	0.892 ± 0.004 *	0.796 ± 0.007 *	8.9 ± 0.3 *	19.36 ± 0.55

**Table 3:** Cross-market generalization results (training on source market, testing on target market).

Source→Target	Method	Precision	Recall	F1-Score	AUC	EWT (min)
NYSE/NASDAQ→SSE/SZSE	Transformer (numerical only)	0.732	0.641	0.683	0.781	3.6
NYSE/NASDAQ→SSE/SZSE	Simple cross-attn fusion	0.768	0.682	0.722	0.824	5.2
NYSE/NASDAQ→SSE/SZSE	Proposed method	0.816	0.731	0.771	0.868	7.6
SSE/SZSE→NYSE/NASDAQ	Transformer (numerical only)	0.721	0.628	0.671	0.769	3.3
SSE/SZSE→NYSE/NASDAQ	Simple cross-attn fusion	0.759	0.674	0.714	0.817	5.0
SSE/SZSE→NYSE/NASDAQ	Proposed method	0.807	0.722	0.762	0.861	7.2

**Table 4:** Module ablation results.

Model Variant	Precision	Recall	F1-Score	AUC	EWT (min)
w/o Cross-modal temporal alignment	0.821	0.742	0.779	0.861	6.4
w/o Multilingual semantic denoising	0.833	0.751	0.790	0.871	7.1
w/o Semantic–numerical collaborative fusion	0.826	0.748	0.785	0.866	6.7
Numerical branch only	0.781	0.706	0.742	0.823	4.7
Full model (proposed method)	0.852	0.781	0.815	0.892	8.9

**Table 5:** Ablation results of different linguistic configurations on sensing performance.

Linguistic Configuration	Precision	Recall	F1-Score	AUC	MCC	EWT (min)
English-only Baseline	0.806	0.746	0.775	0.848	0.742	6.4
Chinese-only Baseline	0.792	0.736	0.763	0.835	0.725	5.9
Multilingual (Proposed)	0.852	0.781	0.815	0.892	0.796	8.9

**Table 6:** Sensitivity analysis of the temporal decay parameter γ on sensing performance.

Decay Coefficient γ	Precision	Recall	F1-Score	AUC	MCC	EWT (min)
γ=0.001 (Slow Decay)	0.786	0.741	0.763	0.832	0.718	9.4
γ=0.01	0.824	0.768	0.795	0.864	0.765	9.2
γ=0.1 (Optimal)	0.852	0.781	0.815	0.892	0.796	8.9
γ=1.0	0.835	0.739	0.784	0.857	0.751	5.6
γ=5.0 (Fast Decay)	0.812	0.684	0.742	0.816	0.698	3.4

## Data Availability

The source code, data preprocessing pipelines, and anonymized feature-level datasets presented in this study will be made publicly available upon the acceptance of the manuscript at https://github.com/Aurelius-04/MSNCformer.git (accessed on 1 April 2026). While the raw records from proprietary sources such as Refinitiv and Bloomberg are subject to licensing restrictions, we provide comprehensive documentation, API templates, and data cleaning scripts within the repository to ensure that the data acquisition and reconstruction process is fully transparent and reproducible for researchers with institutional access to these platforms.

## References

[1] Joshi P., Santy S., Budhiraja A., Bali K., Choudhury M. (2020). The state and fate of linguistic diversity and inclusion in the NLP world. arXiv.

[2] Chen A., Wei Y., Le H., Zhang Y. (2024). Learning by teaching with ChatGPT: The effect of teachable ChatGPT agent on programming education. Br. J. Educ. Technol..

[3] Ousidhoum N., Beloucif M., Mohammad S. (2025). Building Better: Avoiding Pitfalls in Developing Language Resources when Data is Scarce. Proceedings of the 63rd Annual Meeting of the Association for Computational Linguistics (Volume 1: Long Papers).

[4] Ruder S., Vulić I., Søgaard A. (2019). A survey of cross-lingual word embedding models. J. Artif. Intell. Res..

[5] Aharoni R., Johnson M., Firat O. (2019). Massively multilingual neural machine translation. arXiv.

[6] Conneau A., Khandelwal K., Goyal N., Chaudhary V., Wenzek G., Guzmán F., Grave E., Ott M., Zettlemoyer L., Stoyanov V. (2020). Unsupervised cross-lingual representation learning at scale. Proceedings of the 58th Annual Meeting of the Association for Computational Linguistics.

[7] Dal Pozzolo A., Caelen O., Johnson R.A., Bontempi G. (2015). Calibrating probability with undersampling for unbalanced classification. Proceedings of the 2015 IEEE Symposium Series on Computational Intelligence.

[8] Akoglu L., Tong H., Koutra D. (2015). Graph based anomaly detection and description: A survey. Data Min. Knowl. Discov..

[9] Devlin J., Chang M.W., Lee K., Toutanova K. (2019). Bert: Pre-training of deep bidirectional transformers for language understanding. Proceedings of the 2019 Conference of the North American Chapter of the Association for Computational Linguistics: Human Language Technologies, Volume 1 (Long and Short Papers).

[10] Zhang L., Zhang Y., Ma X. (2021). A new strategy for tuning ReLUs: Self-adaptive linear units (SALUs). Proceedings of the ICMLCA 2021; 2nd International Conference on Machine Learning and Computer Application.

[11] Chalapathy R., Chawla S. (2019). Deep learning for anomaly detection: A survey. arXiv.

[12] Zhan X., Kou L., Xue M., Zhang J., Zhou L. (2022). Reliable long-term energy load trend prediction model for smart grid using hierarchical decomposition self-attention network. IEEE Trans. Reliab..

[13] Conneau A., Wu S., Li H., Zettlemoyer L., Stoyanov V. (2020). Emerging cross-lingual structure in pretrained language models. Proceedings of the 58th Annual Meeting of the Association for Computational Linguistics.

[14] Ruff L., Kauffmann J.R., Vandermeulen R.A., Montavon G., Samek W., Kloft M., Dietterich T.G., Müller K.R. (2021). A unifying review of deep and shallow anomaly detection. Proc. IEEE.

[15] Chen T., Kornblith S., Norouzi M., Hinton G. A simple framework for contrastive learning of visual representations. Proceedings of the International Conference on Machine Learning.

[16] Khosla P., Teterwak P., Wang C., Sarna A., Tian Y., Isola P., Maschinot A., Liu C., Krishnan D. (2020). Supervised contrastive learning. Adv. Neural Inf. Process. Syst..

[17] Song A., Seo E., Kim H. (2023). Anomaly VAE-transformer: A deep learning approach for anomaly detection in decentralized finance. IEEE Access.

[18] Feng H., Wang Y., Fang R., Xie A., Wang Y. Federated risk discrimination with siamese networks for financial transaction anomaly detection. Proceedings of the P2025 2nd International Conference on Digital Economy and Computer Science.

[19] Sakurada M., Yairi T. Anomaly detection using autoencoders with nonlinear dimensionality reduction. Proceedings of the MLSDA 2014 2nd Workshop on Machine Learning for Sensory Data Analysis.

[20] Pires T., Schlinger E., Garrette D. (2019). How multilingual is multilingual BERT?. arXiv.

[21] Wu S., Dredze M. (2020). Are all languages created equal in multilingual BERT?. arXiv.

[22] Gururangan S., Marasović A., Swayamdipta S., Lo K., Beltagy I., Downey D., Smith N.A. (2020). Don’t stop pretraining: Adapt language models to domains and tasks. arXiv.

[23] He D. (2025). A multimodal deep neural network-based financial fraud detection model via collaborative awareness of semantic analysis and behavioral modeling. J. Circuits Syst. Comput..

[24] Xu J., Lo S.Y., Safaei B., Patel V.M., Dwivedi I. Towards zero-shot anomaly detection and reasoning with multimodal large language models. Proceedings of the Computer Vision and Pattern Recognition Conference.

[25] Pan X., Wang D., Tsung F. (2025). Empowering Intelligent Quality Control with Large Models: A Comprehensive Survey of Methods, Challenges, and Perspectives. TechRxiv.

[26] Wang Y., Yao Q., Kwok J.T., Ni L.M. (2020). Generalizing from a few examples: A survey on few-shot learning. ACM Comput. Surv. (CSUR).

[27] Wu Y., Xiang C. Multimodal Financial Anomaly Detection in Enterprises Using VAE–Transformer–GNN Hybrid Ensemble Models. Proceedings of the 2nd International Symposium on Integrated Circuit Design and Integrated Systems.

[28] ForouzeshNejad A.A., Arabikhan F., Gegov A., Jafari R., Ichtev A. (2025). Data-driven predictive modelling of agile projects using explainable artificial intelligence. Electronics.

[29] Hu J., Ruder S., Siddhant A., Neubig G., Firat O., Johnson M. Xtreme: A massively multilingual multi-task benchmark for evaluating cross-lingual generalisation. Proceedings of the International Conference on Machine Learning.

[30] Lauscher A., Ravishankar V., Vulić I., Glavaš G. (2020). From zero to hero: On the limitations of zero-shot cross-lingual transfer with multilingual transformers. arXiv.

[31] Xiang Y. (2022). Using Arima-Garch model to analyze fluctuation law of international oil price. Math. Probl. Eng..

[32] Liu F.T., Ting K.M., Zhou Z.H. (2008). Isolation forest. Proceedings of the 2008 Eighth IEEE International Conference on Data Mining.

[33] Chen H., Liu H., Chu X., Liu Q., Xue D. (2021). Anomaly detection and critical SCADA parameters identification for wind turbines based on LSTM-AE neural network. Renew. Energy.

[34] Hewage P., Behera A., Trovati M., Pereira E., Ghahremani M., Palmieri F., Liu Y. (2020). Temporal convolutional neural (TCN) network for an effective weather forecasting using time-series data from the local weather station. Soft Comput..

[35] Han K., Wang Y., Chen H., Chen X., Guo J., Liu Z., Tang Y., Xiao A., Xu C., Xu Y. (2022). A survey on vision transformer. IEEE Trans. Pattern Anal. Mach. Intell..

[36] Chen Y., Wang Q., Wu S., Gao Y., Xu T., Hu Y. (2024). Tomgpt: Reliable text-only training approach for cost-effective multi-modal large language model. ACM Trans. Knowl. Discov. Data.

[37] Gadzicki K., Khamsehashari R., Zetzsche C. (2020). Early vs late fusion in multimodal convolutional neural networks. Proceedings of the 2020 IEEE 23rd International Conference on Information Fusion (FUSION).

[38] Wu H., Sun Y., Yang Y., Wong D.F. (2025). Beyond Simple Fusion: Adaptive Gated Fusion for Robust Multimodal Sentiment Analysis. arXiv.

